# Coping, quality of life, and hope in adults with primary antibody deficiencies

**DOI:** 10.1186/1477-7525-3-31

**Published:** 2005-05-04

**Authors:** Hanne Marie Høybråten Sigstad, Asbjørg Stray-Pedersen, Stig S Frøland

**Affiliations:** 1 Centre for Rare Disorders, Rikshospitalet University Hospital, Oslo, Norway; 2 The Department of Special Needs Education, University of Oslo, Oslo, Norway; 3 Department of Medical Genetics, Rikshospitalet University Hospital, Oslo, Norway; 4 Section of Clinical Immunology and Infectious Diseases, Department of Medicine, Rikshospitalet University Hospital, Oslo, Norway

**Keywords:** primary immunodeficiency diseases

## Abstract

**Background:**

Living with a chronic disease, such as primary antibody deficiency, will often have consequences for quality of life. Previous quality-of-life studies in primary antibody deficiency patients have been limited to different treatment methods. We wanted to study how adults with primary antibody deficiencies manage their conditions and to identify factors that are conducive to coping, good quality of life and hope.

**Methods:**

Questionnaires were sent to all patients ≥20 years of age with primary antibody deficiencies who were served by Rikshospitalet University Hospital. The questionnaires consisted of several standardized scales: Ferrans and Powers Quality of Life Index (QLI), Short Form-36 (SF-36), Jalowiec Coping Scale (JCS), Nowotny Hope Scale (NHS), and one scale we devised with questions about resources and pressures in the past. Of a total of 91, 55 patients (aged 23–76 years) answered the questionnaires. The questionnaire study were supplemented with selected interviews of ten extreme cases, five with low and five with high quality of life scores.

**Results:**

Among the 55 patients, low quality of life scores were related to unemployment, infections in more than four organs, more than two additional diseases, or more than two specific occurrences of stress in the last 2–3 months. Persons with selective IgA deficiency had significantly higher QLI scores than those with other antibody deficiencies. An optimistic coping style was most frequent used, and hope values were moderately high. Based on the interviews, the patients could be divided into three groups: 1) low QLI scores, low hope values, and reduced coping, 2) low QLI scores, moderate hope values, and good coping, and 3) high QLI scores, moderate to strong hope values, and good coping. Coping was related to the patients' sense of closeness and competence.

**Conclusion:**

Low quality of life scores in adults with primary antibody deficiencies were linked to unemployment and disease-related strains. Closeness and competence were preconditions for coping, quality of life and hope. The results are valuable in planning care for this patient group.

## 1. Background

Primary immunodeficiency diseases represent a heterogeneous group of rare disorders characterized by an increased susceptibility to infections and autoimmune diseases. Primary antibody deficiencies (PAD) constitute the largest subgroup and include: Common Variable Immunodeficiency, X-linked (Brutons) Agammaglobulinemia, Selective IgA deficiency, IgG subclass deficiency, and Hyper IgM syndrome [1]. Some patients need lifelong replacement therapy with immunoglobulins and/or frequent courses of antibiotics as treatment and/or prophylaxis. Patients with PAD have increased incidence of auto-immune diseases and experience long-term complications of infections and/or treatment [2]. Living with a chronic disease, such as PAD, will often have consequences for quality of life. Previous quality-of-life studies in PAD patients have been limited to different treatment methods. After initiation of subcutaneous replacement therapy, increased health-related function and improved self-rated health have been reported [3]. We wanted to study wider aspects of quality of life among adults with PAD: How do they manage their condition? Which factors are conducive to coping, good quality of life, and hope?

Coping, quality of life, and hope are important aspects when the effects of a disease from infancy to old age are examined. There are various partially overlapping perspectives on, and definitions of coping, quality of life, and hope [4]. Coping reflects a process and includes active involvement over a period of time [5,6]. Hope and quality of life describe outcomes rather than processes. Hope and quality of life are concepts which have several dimensions. Coping also includes different strategies, but the total sum of the strategies does not constitute a global definition of the concept. Choice of strategies can influence outcome variables such as hope or quality of life positively or negatively. Coping is of importance for quality of life, and hope can be regarded as a coping strategy [7]. Hope can be seen as a variable that positively contributes to the experience of quality of life.

Coping is defined by Lazarus and Folkman [[5]; p.141] as "Constantly changing cognitive and behavioural efforts to manage, reduce or tolerate external and/or internal demands that are appraised as taxing or exceeding the resources of the person". The coping process depends on the situational context in which it occurs [5]. According to Lazarus and Folkman's theory [5,6], resources and pressures are linked to coping. We used resources and pressures as concepts in the present study. Resources can be divided in two groups; personal and socio-ecological resources. Pressures, such as disease-related experiences, may lead to stress and to reduced coping ability.

Locus of control is seen as a crucial factor in coping [8]. An internal locus of control is present when a person explains events by referring to causes within themselves. The person perceives that the event is contingent upon his/her own behavior or his/her own relatively permanent characteristics. An external locus of control is present when a person explains events by referring to causes in the situation or environment. Events and circumstances are typically perceived as the result of luck, chance, fate, under the control of powerful others, or unpredictable because of the great complexity of the forces surrounding the person given an external locus of control.

**Figure 1:**
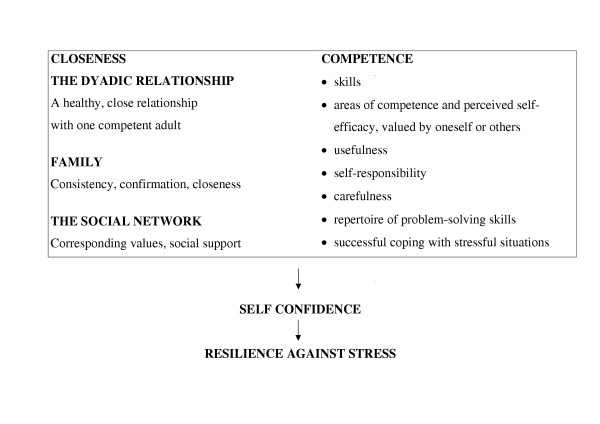
**Closeness and competence: preconditions for resilience against stress**. (Sommerschild, 1998 [11]).

Resilience predisposes for successful coping [9,10]. Longitudinal studies in high risk children have showed positive correlation between resilience and overcoming difficult social circumstances as adolescents. Sommerschild [11] developed a theoretical model which sums up of the key points of resilience theory (see Figure 1). Resilience refers to the person's latent resources which can be mobilized in defense of the self in stressful situations. Resilience is based on self confidence. Closeness and competence contribute to self confidence. Figure 1 shows the concept closeness explained at three levels; the dyadic relationship with one competent adult, family, and the social network. Competence encompasses the person's skills and experience of usefulness.

In the present study, quality of life was defined as a person's overall satisfaction with life: "A person's sense of well-being that stems from satisfaction or dissatisfaction with areas of life that are important to him/her" [[12]; p. 15]. The global concept of quality of life is represented by four domains: A health and functioning domain, a socio-economic domain, a psychological/spiritual domain, and a family domain. Health-related quality of life is defined as a person's satisfaction or happiness within areas of life that are affected by health or health care. Health-related quality of life includes eight domains: physical functioning, role physical, bodily pain, general health perceptions, vitality, social functioning, role emotional, and mental health.

Hope is future-oriented and described as a feeling, an emotion, an experience, a need and a dynamic attribute [13,14]. "A six-dimensional, dynamic attribute of the person which orients to the future includes: active involvement by the individual, comes from within, is possible, relates to or involves others or a higher being, and relates to meaningful outcomes to the individual" [[15]; p.89].

Two questions have been central in the present study:

1. How do adults with PAD manage their condition?

2. What kinds of factors influence their coping, quality of life and hope?

## 2. Methods

The survey was the main investigation in this study, and was analyzed quantitatively. The interviews were included as a supplement to the survey, and did not represent a traditional qualitative study.

### The survey

#### Sample

As of 2000, 122 patients with PAD were registered in Norway [1]. All PAD patients ≥ 20 years and served by Rikshospitalet, in total 91 persons, received the questionnaires, after excluding one cognitively impaired person. The cohort included 50 men and 41 women, aged 20–82 years, with various PAD diagnoses: Common variable immunodeficiency (n = 66), X-linked Agammaglobulinemia (n = 8), Selective IgA deficiency (n = 16) and Hyper IgM syndrome (n = 1).

55 of the 91 adults we approached completed the questionnaires (60% response rate), 31 men and 24 women. The mean age was 41.6 (median 38, range 20–76) years. The sample included 29 young adults, aged 20–39 years and 26 older adults, aged 40–76 years. Distribution of the specific PAD diagnoses was: Common Variable Immunodeficiency (n = 43), X-linked Agammaglobulinemi (n = 3), Selective IgA deficiency (n = 8) and Hyper IgM syndrome (n = 1). Nine of the responders reported previous infection with Hepatitis C virus (HCV). Specific PAD diagnoses, gender, and mean age, 47.2 (44, 20–82) years, were similar and not significant different, when responders and non-responders were compared. Responders (n = 55) were reasonably representative of the original cohort (n = 91).

#### Measures

The survey included questions about the individual's past and present situations, and about his/her thought concerning plans for the future. Demographic variables including age, gender, education, employment and marital status were requested. Information was elicited concerning the following disease-related variables: specific diagnosis, duration of clinical immunodeficiency state, frequency of infections, in which organs the infections (acute and chronic) occurred, other medical complications (including Hepatitis C virus infection), additional diseases (including auto-immune diseases), treatment with subcutaneous (ScIg) versus intravenous immunoglobulin (IVIg), other treatments (antibiotics) and stressful events in the previous 2–3 months. Disease-related strains were defined as the most problematic strains linked to the PAD diagnosis, medical complications or additional diseases.

Five different scales were incorporated into one comprehensive 30-page questionnaire. Four of the standardized scales had previously been translated and tested in Norwegian populations [7,16,17]. The standardized scales were: Ferrans and Powers Quality of Life Index (QLI) [18], Short Form-36 (SF-36) [19], Jalowiec Coping Scale (JCS) [20], and Nowotny Hope Scale (NHS) [13]. An additional scale designed for this project (RPP Scale), focused on resources and pressures in the past. Factor analyses were used to assess the empirical support for each subscale in all instruments. Internal consistency was estimated using Cronbach's alpha coefficient.

*Ferrans and Powers Quality of Life Index (QLI) *has been designed to measure quality of life in both ill and healthy individuals, and this was based on Ferrans' definition of quality of life [12,18]. The global construct quality of life is represented by four underlying subscale domains/subscales:

• Health/Functioning

• Socio-economic

• Psychological/Spiritual

• Family

The QLI consists of two sections. One section measures satisfaction within various domains. The other section measures the importance of each domain for the subject. The items are scored according to a 6-point Likert scale ranging from "very satisfied" to "very dissatisfied" for the satisfaction items, and from "very important" to very unimportant" for the importance items. The overall score is the product of the satisfaction responses and the importance responses. The possible range for the overall and subscale scores is 0–30, the higher the score the better quality of life.

The validity and reliability of QLI has previously been evaluated. Content validity of the original version was assessed on the basis of a review of the literature [12,18]. Concurrent validity of the QLI was provided by a correlation (r = 0.80) between the QLI and a measure of satisfaction with life [12]. Construct validity was found to be satisfactory in different patient populations, and was confirmed by factor analysis ("the maximum-likelihood method" and "the direct oblimin method of rotation") [12,21]. A four-factor solution had the best fit with the data. Internal consistency reliability was 0.95 for the global score, ranging from 0.66 to 0.93 for the subscales. The test-retest reliability varied from 0.87 at two weeks to 0.81 at one month [18].

QLI has been translated and tested in a Norwegian population of newly diagnosed cancer patients [7]. 131 cancer patients participated in the test, 103 in the retest. The Cronbach's alpha coefficient for the QLI was 0.93 for test and 0.95 for retest. The coefficients for the subscales in both tests ranged from 0.79 to 0.91 [7]. Test-retest-reliability was r = .78 within three-four weeks (Pearsons correlation coefficient). The correlation coefficients ranged from r = .65 to r = .83 for the different subscales. Construct validity was analysed by "the maximum-likelihood method" and "direct oblimin method" of rotation (factor analysis). Eight factors had an "Eigenvalue greater than 1". Four factors explained only 45.4% of the variance in this cancer patient cohort, in contrast to 91% in the study of Ferrans and Power [21].

Reliability analyses in the present study with 55 PAD patients showed a Cronbach's alpha of 0.81 for the total QLI, and ranged from 0.54 to 0.92 for the four subscales. The Family subscale had the lowest alpha value, and the Health/Functioning subscale had the highest. Factor analyses based on the four subscales were done by "the maximum-likelihood method", non-rotated method and "direct oblimin method", rotated. Non-rotated method with "Eigenvalue greater than 1", resulted in one cluster where only one of the factors appear, (2.571). All the subscales could be related to this factor. The factor explained 64.3% of the variance in our sample. This result supported a total scale with a common component.

*The Short Form-36 (SF-36)*, one of several generic questionnaires developed to assess health-related quality of life [19], consists of 36 items which measure eight conceptual domains:

• Self-reported General Health (GH)

• Physical Functioning (PF)

• Bodily Pain (BP)

• Mental Health (MH)

• Role limitations (Physical) (RP)

• Role limitations (Emotional) (RE)

• Vitality (VT)

• Social Functioning (SF)

In addition, one item assesses change in health in the past year (HT). The scores in each domain are transformed into 0–100 scales. The higher score the better health-related quality of life.

The reliability of the eight scales has been estimated using both internal consistency and test-retest methods [22]. Reliability coefficients for each of the eight scales were equal or greater than .80 (ranging from .81 in General Health to .93 in Physical Functioning) with the exception of Social Functioning, which had a reliability of .68. The content validity of the SF-36 has been compared to that of other widely used generic health surveys. Systematic comparisons reveal that the SF-36 includes eight of the most frequently represented health concepts.

SF-36 has previously been translated and tested in 2323 persons from the general Norwegian population [17,23]. Reliability analyses (Cronbach's alpha) showed values from 0.80 to 0.93 for the eight subscales, Role limitations (Emotional) had the lowest and Bodily Pain the highest value. Correlations between the SF-36-scales ranged from r = .29 (Mental Health and Physical Functioning) to r = .68 (Mental Health and Vitality).

Reliability analysis (Cronbach's alpha) of the SF-36 in the present study (n = 55) yielded values from 0.74 to 0.92, Role limitations (Emotional) had the lowest and Social Functioning the highest value. Factor analysis is not usually performed when using SF-36. In spite of a relatively small sample size, factor analysis was done in this study by "Principal Component Analysis", non-rotated method and "Varimax with Kaiser Normalization" rotated method of the eight subscales. The analyses revealed two main factors. The scales which contributed the sum score of Physical Health, were one factor. The scales which contributed the sum score of Mental Health, constituted the other factor. Compared with the original SF-36, there was one finding of note in the present study: the Social Functioning subscale was correlated with the sum score of Physical Health. The Social Functioning subscale in the SF-36 is originally included in the sum score of Mental Health.

*The Jaloviec coping scale (JCS) *is based on Lazarus and Folkman's theory of stress and coping [5,6,20]. The JCS has been designed to measure how people cope with various types of physical, emotional and social stressors. The JCS measures the use and effectiveness of 60 cognitive and behavioural coping strategies in a stressful situation. The items describe cognitive and behavioural efforts in response to stress. In our questionnaire, stress was specified as stress induced by living with PAD. The strategies are grouped into eight coping dimensions:

• Confrontive – "tried to change the situation"

• Evasive – " put off facing up to the problem"

• Optimistic – "tried to think positively"

• Fatalistic – "accepted the situation because very little could be done"

• Emotive – "worried about the problem"

• Palliative – "tried to keep busy and work harder"

• Supportive – "depended on others to help out"

• Self-reliant – "preferred to work things out yourself"

Item responses are rated on a 4-point scale from 0 (never used) to 3 (often used), and a scale of helpfulness from 0 (not helpful) to 3 (very helpful). The higher score, the more coping effort involved. The higher total coping score the more alternation between different coping strategies.

The JCS has previously been tested in several studies [20,24]. Its content validity has been assessed by an expert panel and is supported by a broad theoretical and empirical base. Construct validity has been evaluated. The 60 items are classified into eight subscales, with an agreement ranging from 94% on the Supportive subscale to 54% on the Emotive subscale. Reliability of the JCS, assessed with Cronbach's alpha coefficients and based on results from 24 different studies ranged from 0.48 to 0.81 for the use subscales and from 0.48 to 0.82 for the effectiveness subscales.

JCS has previously been translated and tested in a Norwegian population of 273 patients with psoriasis [16]. Correlations between the eight subscales in JCS ranged from r = .39 (p < .001) to r = .73 (p < .001). Reliability analyses (Cronbach's alpha) of the eight subscales ranged from 0.55 to 0.88. The construct validity of the JCS was analysed by "Principal Component Analysis" with orthogonal rotation (factor analysis). The analyses resulted in three coping dimensions with sufficient internal consistency: confrontive problem-solving coping, normalizing / optimistic coping and combined emotive engagement. 37 % of the total variation in the Norwegian version of JCS was attributed to these three factors [16].

Because few patients responded to the coping effectiveness part of the JCS in the present study, we have only included the coping strategy use part. Reliability analyses (Cronbach's alpha) ranged from 0.41 to 0.75 for the eight subscales (use-scores). The Confrontive, the Evasive and the Optimistic subscales yielded the highest values from 0.73 to 0.75, and the Fatalistic subscale the lowest alpha at 0.41. Our finding of three subscales with the strongest internal consistency is in keeping with the results of Jaloviec et. al. [20]. Factor analyses based on subscales, done by "Principal Component Analysis", non-rotated method and "Varimax with Kaiser Normalization", rotated method, resulted in a three-factor-solution with factor 1: Evasive, Fatalistic and Self-reliant coping; factor 2: Confrontive, Emotive, Palliative coping; and factor 3: Optimistic coping. These analyses revealed that the Optimistic scale could be considered a separate contributing factor in the present study.

*Nowotny Hope Scale (NHS) *is designed to measure hope in a general adult population after a stressful event [13,15], and has been employed primarily in cancer patients. NHS is a 29-item scale with items scored on a 4-point Likert Scale ranging from 4, strongly agree, to 1, strongly disagree. It consists of six subscales:

• Confidence

• Relates to others

• Future is possible

• Spiritual beliefs

• Active involvement

• Comes from within

The total score range is from 29 to 116, with a high score indicating high hope. Cut-off scores are developed for four levels of hope.

The content validity of the NHS has been evaluated by an expert panel [13]. The concurrent validity was established with the Beck Hopelessness Scale (r = -.47). The construct validity of NHS was analysed by "Principal Components Analysis" with orthogonal rotation (factor analysis). The result supported the six dimensions and subscales of hope. Cronbach alpha's reliability coefficient for this instrument was 0.90. The concurrent validity has been found to be satisfactory [13].

NHS has been translated and tested in the above-mentioned Norwegian population of newly diagnosed cancer patients [25]. Correlations between different subscales ranged from r = -.16 to r = .73. Factor analysis done by "Principal Components Analysis", showed that the items of the "Spiritual beliefs" subscale appeared as one factor, and the "Comes from within" items as another factor analogous to Nowotny's subscale items. With the exception of these two factors, the results of the NHS factor analysis of Rustøen [25] diverged from Nowotny's original six dimensions. At three-four week test-retest of NHS correlation was high, Pearson's r = .81. Correlation coefficients ranged from r = .59 to r = .92 for the various subscales. Cronbach's alpha for NHS was 0.89 both in the test and the retest. The alpha coefficients for the subscales ranged between 0.53 and 0.95.

Reliability analysis of NHS in the present study (n = 55) showed a total Cronbach's alpha of 0.87. Cronbach's alpha of the six subscales ranged from 0.54 to 0.94. Our results were similar to Rustøen and Moum's results [25]. Factor analyses based on subscales were done by "Prinicipal Component Analysis", non-rotated method, and "Varimax with Kaiser Normalization", rotated method. Both the non-rotated and the rotated variant of factor analysis seemed to confirm only two explicit contributing factors: factor 1; the Spiritual subscale, and factor 2; the five other subscales.

*The Resources and Pressures in the Past Scale (RPP Scale) *was theoretically founded on Lazarus and Folkman's theory of coping [5,6] and consisted of 64 items divided into two main categories: Resources and Pressures. The past was defined as the period from adolescence to present time. The distinction between different domains within the person's resources was based on previous studies of coping [9,10,26-28]. The concept of Resources included both personal characteristics / temperament and social support resources. Pressures were defined as the person's individual perception of his/her experiences, including immunodeficiency-related experiences and general events.

Resources consisted of four subscales:

• Personal characteristics / temperament

• Family and supporting adults

• Supporting persons in school and social network

• Public Health Service

Pressures in our scale consisted of four subscales:

• Immunodeficiency-related events (for example: many hospitalizations because of the disease)

• General events

• Immunodeficiency-related experiences in school (for example: significant absence from school because of the disease)

• General experiences in school

The items were scored on a 5-point Likert Scale ranging from 5, strongly agree, to 1, strongly disagree. The total score range of Resources was from 29 to 145, and the total score range of Pressures was from 35 to 175. A high score indicated either a good availability of resources or a high level of strain.

In the present study, missing was handled by the same procedure as in the standardized scales JCS and NHS: when more than 50% of the subscale is answered, the missing value is replaced by the mean score of the rest of the subscale.

This scale was evaluated by reliability analyses (Cronbach's alpha) and factor analyses. The aim behind using reliability analyses was to evaluate in what degree the individual items correlated with the main concepts in the scale. Some items were excluded to attain highest possible consistency. The Cronbach's alpha was 0.89 for Resources and 0.90 for Pressures, with a range from 0.63 (Personal characteristics/temperament) to 0.90 (Family and supporting adults) in Resources, and a range from 0.59 (Immunodeficiency-related events) to 0.89 (Immunodeficiency-related experiences in school) in Pressures. Validity was tested by "Principal Component Analysis", non-rotated method and "Varimax with Kaiser Normalization" with rotation (factor analysis) based on the subscales. With "Eigenvalue greater than 1", the non-rotated method made only one contributing factor appear for the Pressures scale to which 55% of the total variance could be attributed. Likewise, the factor analysis (non-rotated method) yielded only one contributing factor from the Resources' scale, to which 46% of the total variance could be attributed.

#### Statistical analyses of the survey data

The SPSS-PC statistical program (v 9.0) was used for data analyses. Descriptive analyses were performed to assess the characteristics of the sample. The impact of demographic and clinical variables on the dependent variables was assessed by t-test for independent samples (two-tailed). The effect sizes were measured by the difference between the means of the samples divided by the mean of the standard deviations of the samples [29]. The effect size was defined by qualitative standardized values (small = .25SD, medium = .50SD, large = 1.00SD). Both correlation analyses and multiple regression analyses were performed to assess the relationship between variables.

### The interviews

The interviews were included as a supplement to the survey to elucidate preconditions for coping, good quality of life, and hopefulness. The interview study was designed to probe and to aid in the interpretation of some of the results from the questionnaire. Cases were selected for interviews to detect possible patterns within two groups: patients with high QLI scores and patients with low QLI scores. The selected cases represented a strategic sample of patients with the lowest and highest QLI scores (n = 21). Originally, we wanted to interview all these extreme cases (n = 21). Ten patients consented to participate in the interview study. Ten cases were regarded as sufficient to detect patterns within the different groups. The qualitative interviews were based on significant results related to QLI in the survey. The interviews were semi-structured with a pre-written interview guide, and lasted nearly two hours.

The interview guide was based on the most central issues in the survey: previous resources and pressures, the interviewees' experience of coping and quality of life, and their hope for the future. Questions about coping included present challenges and choice of coping strategies. Questions about experience of quality of life were related to the four dimensions in QLI: Health/Functioning, Socio-economic, Psychological/Spiritual and Family. Questions concerning hope, dealt with the patients' general experience of hope. All concepts were related to a lifespan perspective as the interviewees were asked to evaluate their present situation related to their past. All interviews were done by the same person and tape-recorded.

In accordance with Kvale's methodology [30], the interviews were analyzed on a thematic and a theoretical level. Kvale distinguishes between three different contexts of interpretation of the interview statements. The thematic level implies a condensed form of what the interviewees themselves understand to be the content of their statements. The interpretation is more or less based on the interviewees' self-understanding as understood by the researcher. The common sense level represents a critical common sense understanding. The interpretations may include a wider frame of understanding than those of the interviewees themselves. The interviewer should be critical of what is said, and may focus on the content of the statement. The theoretical level is a framework for interpreting the meaning of a statement. These interpretations are likely to go beyond the interviewees' self-understanding and to exceed common sense understanding. In this study, the theoretical level included the common sense understanding.

The interviews were analyzed to identify interesting and important themes. New themes appearing during the interviews were included in the analyses (thematic level). Similarities and differences were described within and between the extreme groups. The expressed meanings were summarized into shorter terms. Individual texts were further analyzed with respect to meaning of the texts, and to their respective categories. The categories were divided into groups defined by contrasting elements within and across the groups denoting high and low quality of life [31]. When thematic analyses showed differences within or between the extreme groups, further analyses were done. Since some of these variants seemed to be in accordance with previous studies in coping research, the results of the first thematic analyses were reanalyzed according to relevant theory about the constructs of coping, quality of life, and hope (theoretical level).

According to Kvale [30] reliability pertains to the consistency of the research findings, and validity to the truth and correctness of a statement. Kvale emphasizes that issues of verification do not belong to a separate stage of an investigation, but should be addressed throughout the entire process. Validation is done at seven stages in the interview process: 1. *Thematizing *based on the logic of derivations from theory to the research questions of the study, 2. *Designing *dependent on the adequacy of the design and the methods used for the purpose of the study, 3. *Interviewing *based on trustedness of the interviewees reports and the quality of the interviewing itself, 4. *Transcribing *dependent on the quality of the translation from oral to written language, 5. *Analyzing *dependent on whether the questions in the interview text are valid and whether the interpretations are logical, 6. *Validitating*, based on reflective consideration of what forms are relevant to a specific study and 7. *Reporting *dependent on to what degree a given report is a valid account of the main findings of a study.

## 3. Ethical aspects

The study was approved by the Norwegian Regional Committee for Medical Research Ethics and the Norwegian Social Science Data Services. Participants were guaranteed anonymity and the right to withdraw from the study at any time. An information letter to respondents provided information about the potentially sensitive items.

## 4. Results

### The survey

t-tests were done on selected demographic and disease-related variables and the results are presented in table 1(see Additional file 1). Other results and comparisons are only presented in the text. Table 1 includes results of total scores (means and standard deviations) of all scales in the present study with one exception: the most significant differences in SFscores were found in four out of the eight domains. Only these are presented in the table (BP, MH, RP and SF).

On the RPP, the 55 adults with PAD reported good availability of *resources *in the past (personally and support from others) (mean 3.7, range 2.03–4.93, out of a possible total score of 5). Parents and other supporting adults had been of major importance as social support (mean 3.8, range 1.69–5.00 out of a possible score of 5). Adults with PAD had experienced moderate *pressures *(2.6, 1.27–4.66, out of a possible total score of 5). Pressures related to their immunodeficiency were the most burdensome in the school context (3.09, 1.00–5.00). Four conditions in present time implicated significantly more pressures in the past: younger age (20–39 years) (p = .024), (Effect Size (ES) = .77SD), living alone (p = .015), (ES = .84SD), having more than two additional diseases (p = .005), (ES = 1.07SD), or suffering from infections in more than four organs (p = .038), (ES = .69SD) (t-tests, two-tailed) (Table 1).

The mean score in global QLI (*quality of life*) was moderate at 20.0 (range 12.3–27.6, out of a possible total score of 30.0). In addition to immunodeficiency, the following conditions were associated with significantly lower QLI scores: unemployment (p = .008), (ES = .80SD), infections in more than four organs (p = .020), (ES = .79SD), the presence of more than two other diseases (p = .001), (ES = 1.55SD), or more than two specific occurrences of stress in the last 2–3 months (p = .007), (ES = 1.15SD) (t-tests, two-tailed). These results are presented in table 1. Unemployed men had lower QLI scores compared to employed men (p= .020). The term "unemployed" was defined to include currently/previously unemployed, never employed and recipient of disability pension. Men working full-time achieved significantly higher QLI scores than men working part-time or unemployed men (p = .016). These differences did not exist among the women. Variables without impact on QLI were: length of education, type of treatment (subcutaneous Ig versus intravenous Ig), frequency of treatments, self-administration of treatment at home (ScIg), hospital based treatment (IVIg), and HCV infection.

Compared to a Norwegian sample of newly diagnosed cancer patients (n = 131) [7], the PAD patients (n = 55) had a significantly lower total QLI score (p < .05), along two dimensions: Health and Functioning (p < .05) and Socio-economic (p < .01).

*Health-related quality of life *in different conceptual domains on the SF-36, revealed that the adults with PAD had their lowest mean score in General Health (37.8, range 5.0–87.0, of a possible total score of 100.0) and their highest mean score in Physical Functioning (81.1, 10.0–100.0, of a possible total score of 100.0) (SF-36). The most significant differences in SF scores were found in four of the eight domains, and these are presented in table 1. Gender, employment and disease-related pressures/strains had significant influence. Men had a significantly higher score than women in Bodily Pain, Social Functioning and Vitality, respectively (p = .029), (ES = .64SD); (p = .016), (ES = .68SD); and (p = .004), (ES = .85SD) (t-tests, two-tailed). Unemployed men and women, had significantly lower health-related quality of life, compared to employed adults. Low health-related quality of life was found in Bodily Pain (p = .006), (ES = .81SD); General Health (p = .002), (ES = .88SD); Mental Health (p = .021), (ES = .68SD); Physical Functioning (p = .001), (ES = 1.01SD); Role limitations (Physical) (p = .000), (ES = 1.16SD); and Social Functioning (p = .004), (ES = .91SD). The disease-related strains were infections in more than four organs, infections more than eight times yearly, more than two other diseases and/or more than two specific occurrences of stress in the last 2–3 months. Hepatitis C infection did not have a negative influence on health-related quality of life.

Compared to a control group with a normal distribution (n = 2323) [17], our PAD patients (n = 55) showed significantly lower functional ability scores in all areas of health-related quality of life (SF-36). The finding reached statistical significance (.001) in four areas: General Health, Role limitations (Physical), Social Functioning and Vitality. Compared to a sample of psoriasis patients (n = 283) [4], these PAD patients showed different scores in two areas (SF-36): Bodily Pain, where adults with PAD scored higher (p < .01), and General Health, where adults with PAD scored lower (p < .001).

Of the eight coping strategies measured by the JCS, an optimistic coping strategy was most frequently used (mean item rating 2.28, range 0.44–3.00, on a 4-point scale of 0–3). A palliative coping strategy was rarely used (1.18, 0.20–2.29). The total score for all coping strategies used showed a mean item rating of 1.64 (1.13–2.32). This reflects the extent of use of all coping strategies measured [20]. Being unemployed was associated with high coping scores among adults with PAD (p = .013), (ES = .78SD) (t-tests, two-tailed). Full-time employment was associated with lower coping scores compared to part-time employment, housework or unemployment (p = .021), (ES = .67SD) (Table 1).

The PAD patients had moderate hope values on the NHS with a mean score of 84.9 (range 52–102, of a possible total score of 116). Having more than two additional diseases in addition to PAD was associated with a lower hope value among responders (p = .015), (ES = 1.02SD) (t-tests, two-tailed). The results are presented in table 1. There was positive correlation between being hopeful about the future and quality of life (QLI) in the present, r = .454 (p < .001) (Pearson correlation). Regression analysis with quality of life (QLI) as the dependent variable and hope (NHS) as one of the independent variables, showed R^2 ^= .206 (p < .001), which suggests that hope explains 20.6% of the total variance in the quality of life.

Compared to a sample of newly diagnosed cancer patients (n = 131) [7], our PAD patients (n = 55) had a significantly lower total hope value (p < .05) visualized in two dimensions of NHS: Relates to others (p < .01), and Future is possible (p < .05).

### The interviews

During thematic analysis of the ten interviews, certain categories appeared to be of particular importance. These categories were used as the main categories in the theoretical analysis: quality of life, closeness and competence as resilience, locus of control, and hope. The five responders with high QLI scores showed more homogeneous results than the five responders with low QLI scores for the interview themes resources and pressures in past, coping ability, quality of life, and hope for future. The latter group was split into two subgroups based on criteria related to experience of closeness and competence, and locus of control. Locus of control was determined by evaluating the responders' answers about their own experience of internal control over external occurrences. In spite of a low QLI score, three of the responders seemed to have strong resilience combined with an internal locus of control.

Based on the theoretical analysis, the subjects with a low QLI score were divided into two groups (Group 1 and Group 2). The subjects with a high QLI score were defined as Group 3. Persons in Group 1 (n = 2) had low scores in all four subscales of QLI (Health/Functioning, Socio-economic, Psychological/Spiritual, Family). They had problematic psychological bonds to their mothers, and less experience of closeness or/and competence (Fig 1). They experienced difficulties in coping (self-reported), they had low hope values and either an internal or an external locus of control. In addition, persons in Group 1 had needed various forms of social support. However, they expressed reluctance to receive such support. Persons in Group 2 (n = 3) had low scores in two subscales of QLI: Health/Functioning and Socio-economic. They had especially close relationship to their mothers, but a positive experience of closeness and competence. They were coping successfully (self-reported), had a moderate hope values and an internal locus of control. The persons in Group 2 also needed additional social support, but received such help according to their own wishes. Persons in Group 3 (n = 5) had high scores in all four subscales of QLI. They had experienced closeness and competence, and they were coping successfully. They had moderate to strong hope values, an internal locus of control, and reported no need for additional support.

## 5. Discussion

The purpose of the present study was to study how adults with PAD manage their condition and to identify factors that are conducive to coping, good quality of life, and hopefulness. Low scores in quality of life were linked to unemployment and disease-related strains among adults with PAD. Closeness and competence were preconditions for coping, good quality of life and hope.

The survey showed that parents and other supporting adults were the most important caregivers (*Resources*) in adolescence. This is in accordance with findings in previous coping studies [32]. Not surprisingly, the interviews confirmed the family as the best caregivers during childhood. In cases where the parents did not fulfill their function as caregivers, other people such as neighbors and health personnel functioned as caregivers. In addition, social support was not only associated with positive experiences among the responders with low QLI score. Those with a low QLI score reported a complicated relationship to their mothers. They wanted to be accepted as adults, but did not experience that they were.

Experiences related to immunodeficiency (*Pressures*) were of major importance, for example: episodes of illness, absence from school, psychosocial consequences of the disease, self-respect and respect from other people. These results came from the survey. The interviews confirmed that occurrences related to the immunodeficiency were the most chronic problems. This is in accordance with Ogden's conclusions [33]. Ogden classified painful school experiences as a long-term element of risk. Many studies have emphasized the importance of the impact of previous pressures on later development [32,34-36]. In accordance with their findings, the results of the present study point to previous resources and pressures as crucial factors for future coping ability and maturation.

A high degree of immunodeficiency-related strain as well as unemployment had a negative impact on Health and Functioning on the QLI in this sample (n = 55) (*Quality of life*). In order to achieve a high total QLI score, a low score in one dimension has to be compensated by higher scores in the other dimensions. To be satisfied with one's own achievement as an experience of coping is seen as crucial to achieving a high quality of life [37]. Consequently, unemployment requires that one is able to compensate for lack of employment with another meaningful activity. This may be interest in interpreting the finding in this study that unemployed men reported lower quality of life than men with a steady job.

Persons with Selective IgA-deficiency achieved a higher global QLI score than other PAD patients. Patients with symptomatic selective IgA deficiency are usually healthier than other PAD patients [2]. Surprisingly, the QLI differences were not found in the health/function domain, but in the socioeconomic, family and psychological/spiritual domains.

We observed a difference in QLI between those who treated themselves (ScIg) compared to those who were treated by others (IVIg), but the difference was not statistically significant. However, the PAD patients who treated themselves (ScIg) had a significantly higher score in Social Functioning (SF-36) compared to the others. SF-36 measured *health-related quality of life*. Our study focused on global quality of life, not specific in relation to treatment, and the results did not elucidate all aspects of these different treatment methods. Gardulf [3] found a significantly increased health-related function, and improved self-rated health among patients with PAD after initiation of ScIg infusions. However, our study was not designed to detect differences before and after introduction of a specific treatment method.

Nine of the 55 responders had experienced Hepatitis C virus infection due to contaminated IVIg [38]. Surprisingly, HCV infection had no influence on the QLI scores or scores of SF-36 in this study.

The interviewees with a low QLI score were in poor health and reported some limitations in daily life functioning. Still, these patients showed obvious differences within the group related to other quality of life conditions, as some of them (Group 2) seemed to fully adjust their experience of quality of life. Well-being and satisfactory social support were reported. This was not anticipated because of low QLI scores from the questionnaire. Wilson and Cleary's research studies [39] suggest that there is no direct correlation between serious limitation in health and loss of quality of life. On the other hand, positive self-esteem, ability to be active and to use one's abilities are elements of crucial significance [37,40,41] in achieving a high quality of life score. In our interview study, there was a lack of such characteristics in Group 1 who had low scores in all domains of the QLI. Insufficient involvement, dependence on others, low self-esteem, and lack of happiness and well-being were characteristic interview responses in this group.

There were some differences between the findings in the survey and the interviews: The global QLI scores and SF-scores did not give a good description of social network. Interviews indicated that a good network was important for resilience. The interviews may have described more comprehensively the responders' experience of different types of support. Mutual relationships were identified as important by interview, but had not been included among the specific items in the survey. Psychological characteristics, external living conditions and relationships have been evaluated by others [37] as essential factors for quality of life. The responders with a low QLI score in Group 2 (the interview study) confirmed that a good social network had contributed to increasing their quality of life.

Compared to a Norwegian sample of newly diagnosed cancer patients [7], our 55 PAD patients had a lower global QLI score, along dimensions: Health and functioning and socio-economic. By nature of their disease, the PAD patients have had a life-long course, in contrast to the cancer patients. Perhaps the chronicity of PAD explains some of the divergence.

Compared to the cancer cohort [17], our PAD patients scored lower on functional ability in all areas of health-related quality of life (SF-36). Other quality-of-life studies confirm significant negative implications of physical health limitations [42,43]. Compared to a sample of psoriasis patients [16], our PAD patients scored differently in two areas (SF-36): Bodily Pain, where adults with PAD had a higher score, and General Health, where adults with PAD had a lower score. Bodily pain is not characteristic for PAD patients and can be a possible explanation for the high score in SF-36. Adults with psoriasis have better general health, compared with adults with PAD.

An optimistic *coping *strategy was most frequently used in dealing with the illness (survey). This is a consistent finding in other studies of groups with other chronic diseases [16,44-46]. Employment is linked to competence and may predispose for successful coping (Fig 1) [11]. According to Sommerschild [11], coping depends on competence in various areas, e.g. perceived self-efficacy, usefulness, and problem-solving skills. Unemployment requires that one is able to compensate in other spheres in order to achieve a feeling of competence [32]. Competence and closeness are areas which may be amenable to psychosocial interventions aimed at increasing quality of life in adults with PAD. Prospective interventions can be designed for patients with low quality of life scores or may include all PAD patients.

The PAD patients had moderate *hope *scores on the NHS. Having more than two other diagnoses in addition to PAD was associated with a lower hope score. Less hopefulness correlated positively with a high degree of disease-related strain. We found a definite positive correlation between being hopeful about the future and quality of life (QLI) in the present. Hope seemed to be responsible for 1/5 of the total variance in the quality of life. Increasing hope may have an impact on enhancing quality of life.

Compared to the cohort of newly diagnosed cancer patients [7], our PAD patients had a significantly less global hope score in two dimensions of NHS: "Relates to others", and "Future is possible". Studies confirm that persons with a cancer diagnosis, thought to be in a hopeless situation, often have a positive and hopeful vision of the future [47]. The PAD patients include people with a congenital chronic disease for which there is no curative treatment. All of the newly diagnosed cancer patients had been diagnosed within the previous year. They were aware that cancer is a terminal illness, but they may have retained hope for curative treatment.

Another study, which focused on sources of hope among people with chronic diseases, emphasized hope as a main coping strategy [48]. Evangelista [49] found that hope strongly correlated with quality of life in a cohort of female heart transplant recipients. The Group 2 and Group 3 patients in our interviews had plans and dreams, and were optimistic about the future, and their plans were related to other people. But in Group 1, thoughts about the future were characterized by fatalism, less faith in the future and lack of involvement with others.

The low response rate (60%) may be considered a limitation of the external validity of the survey. Including subjects with other immunodeficiency disorders would have increased sample size at the cost of homogeneity. Homogeneity was chosen to enhance validity. Simple statistical analyses were employed. We were able to use the t-test after controlling for statistical assumptions (independent samples, normal distribution, and homogeneity of variance). The findings reported as significant reached, with one exception, levels of significance of .01 and .001. Effect size varied between moderately large to a large [29]. In spite of some concerns regarding statistical power, we consider that our results have relatively good *statistical validity *[50].

Five scales were used; four of them standardized and well tested by others, and in Norwegian populations. One scale was specially designed for this study. Seen in a lifespan perspective, related to previous pressures/strains and availability of resources, we showed that the results of this scale also were important for the evaluation of the results from the other scales. In spite of some variation between the subscales, the five scales showed good reliability generally (Cronbach's alpha) in keeping with the main constructs of the study. The four standardized scales were internally consistent and test-retest reliability was satisfactory. Despite a thorough testing of the RPP Scale, the construct validity of this scale may be more uncertain. Nevertheless, in spite of some limitations, the total construct validity appears robust.

If the group is too special, it may not be correct to generalize the results to other kinds of persons or other diseases. However, this sample was characterized by a wide age span (23–76 years), different subgroups of PAD, various types of treatment, age at diagnosis and number of disease-related strains. Analysis of the non-responders showed that the responders in this survey (n = 55) were representative for the entire cohort (n = 91), and they constitute 75% of all patients registered with PAD in Norway [1]. Therefore, this survey was has relatively good *external validity *relative to this patient group.

The results of the interviews were evaluated in according to Kvale's methodology [30] for qualitative research interviewing, and the reliability and validity were found to be reasonably good. An evaluation during the analysis process of the interviews concluded that the size of the sample was sufficient to detect different patterns in these two groups of extreme cases. And similar patterns have been found in previous studies on coping [9,10,51]. Such a confirmation is expected to strengthen the results, and to some extent compensate for the limited sample size. However, the low number necessitates a tentative and explorative use of the study findings.

Since the sample of the ten interview subjects was in accordance with the strategic sample concerning the most central variables impacting on quality of life, *the generalizability *of the interview study could be evaluated as relatively good.

The interviews were included to elucidate nuances in the knowledge from the survey results. Triangulation of different methods is considered an appropriate strategy for strengthening the validity of research findings [52,53]. The interview study supplemented the survey, supported the constructs in the survey and, as a result, strengthened the validity of the findings. In this project, the results from the survey were thoroughly examined during the interviews. However, the interview study also raised some new hypotheses, which were not registered in the survey. The interviews have added new aspects, and functioned as a supplement to the survey, increasing the relevance of the project and its results. Combining the findings from both the survey and the interviews strengthens the validity of the project's end result.

There may be ethical concerns when inviting patients to complete questionnaires about coping, quality of life and hope. In this study, the information letter gave an opportunity to prepare for potentially sensitive questionnaire items. Moreover, the interviews made ethical demands on the interviewer. In an interview, the interviewer is a part of the method, and has a responsibility to manage separating roles, having focus on the interview and taking care of the interviewees in a professional way. In the present study, to get the interviewees' confidence, the interviewer had to define the content of the interview precisely, and explain the interviewers' role. The interviewer referred the interviewees to other health personnel when needed.

## 6. Conclusion

Low scores in quality of life were linked to unemployment and disease-related strain among adults with PAD. Coping was closely linked to the patients' sense of closeness and competence. The results are in accordance with previous studies of other groups with chronic diseases. Closeness and competence are areas where psychosocial interventions may contribute to better quality of life in adults with PAD. The findings are relevant also for other groups of patients. Medical interventions should reduce the patient's strain, and support his or her ability to be employed.

## Supplementary Material

Additional File 1 Table 1. Mean scores (SD) in PAD patients on resources, strains, quality of life, functioning, coping, and hope Click here for file
