# Supplementary material for: Coping, quality of life, and hope in adults with primary antibody deficiencies
Source: Health Qual Life Outcomes. 2005 May 4;3:31. doi: 10.1186/1477-7525-3-31 (PMC1177979; doi:10.1186/1477-7525-3-31)
Supplement: Additional File 1 — Table 1. Mean scores (SD) in PAD patients on resources, strains, quality of life, functioning, coping, and hope [file 1477-7525-3-31-S1.doc]

**Table 1. Mean scores (SD) in PAD patients on resources, strains, quality of life, functioning, coping, and hope**

| **Scales** |  | **RPP** | | **QLI** | **SF-36** | | | | **JCS** | **NHS** |
| --- | --- | --- | --- | --- | --- | --- | --- | --- | --- | --- |
|  | n | *Resources* | *Pressures* |  | *Bodily Pain* | *Mental Health* | *Role limitation (Physical)* | *Social Functioning* |  |  |
| **All responders** | 55 | 3.7 (0.6) | 2.6 (0.6) | 20.0 (3.5) | 66.0 (27.7) | 73.0 (18.6) | 56.5 (42.7) | 70.8 (28.8) | 1.6 (0.3) | 84.9 (9.9) |
| **Age groups**  >39 years  <39 years | 26  29 | 3.6 (0.7)  3.7 (0.6) | 2.4 (0.6)  2.8**^*^** (0.6) | 20.6 (3.3)  19.4 (3.7) | 65.3 (31.4)  66.5 (24.4) | 75.3 (18.0)  71.0 (19.2) | 62.0 (43.4)  51.7 (42.2) | 76.0 (30.4)  66.1 (27.0) | 1.6 (0.3)  1.7 (0.3) | 86.3 (9.6)  83.3 (10.2) |
| **Gender**  Men  Women | 31  24 | 3.6 (0.7)  3.7 (0.6) | 2.6 (0.7)  2.6 (0.5) | 20.5 (3.6)  19.3 (3.4) | 73.6 (21.8)  56.4^*^ (31.6) | 75.7 (19.5)  69.5 (17.0) | 62.5 (40.3)  49.0 (45.1) | 79.2 (23.8)  60.4^*^ (31.6) | 1.6 (0.3)  1.7 (0.3) | 85.1 (10.5)  84.5 (9.2) |
| **Diagnosis**  Selective IgA deficiency  Other immunodeficiencies | 8  45 | 3.8 (0.6)  3.7 (0.6) | 2.5 (0.3)  2.6 (0.6) | 22.5 (2.4)  19.6^*^ (3.6) | 62.3 (29.7)  67.1 (26.5) | 80.5 (18.7)  71.0 (18.4) | 71.9 (26.8)  54.0 (42.8) | 68.8 (35.4)  71.9 (26.8) | 1.8 (0.3)  1.6 (0.3) | 89.7 (7.4)  84.3 (10.3) |
| **Number of infected organs**  0-4 organs  > 4 organs | 46  9 | 3.8 (0.5)  3.2 (0.9) | 3.0 (0.8)  2.5^*^ (0.5) | 20.4 (3.4)  17.7^*^(3.4) | 69.5 (26.2)  48.3^*^(30.1) | 74.5 (17.4)  65.8 (23.5) | 61.1 (41.8)  33.3 (41.5) | 75.8 (24.3)  45.8^*^ (37.5) | 1.6 (0.3)  1.7 (0.2) | 85.9 (9.3)  80.0 (11.7) |
| **Number of infections**  >8 yearly  0-8 yearly | 18  35 | 3.7 (0.5)  3.6 (0.7) | 2.8 (0.6)  2.5 (0.6) | 19.2 (3.6)  20.7 (3.3) | 64.2 (27.0)  68.2 (28.4) | 71.2 (18.9)  75.4 (17.8) | 38.9 (39.5)  67.1^*^ (41.0) | 61.8 (30.1)  77.1 (26.7) | 1.7 (0.3)  1.6 (0.3) | 87.2 (8.5)  83.8 (10.4) |
| **Number of other diseases**  0-2  >2 | 47  8 | 3.8 (0.6)  3.1^**^(0.6) | 2.5 (0.6)  3.2^*^(0.6) | 20.6 (3.4)  16.4^***^(2.0) | 70.6 (26.1)  39.3^**^(28.9) | 76.4 (17.8)  53.5^***^(8.3) | 61.4 (42.1  28.1^*^(36.4) | 77.4 (24.2)  32.8^***^(24.0) | 1.6 (0.3)  1.7 (0.3) | 86.3 (9.2)  76.5^*^(10.0) |
| **HCV infection**  Yes  No | 9  44 | 3.5 (0.8)  3.7 (0.6) | 2.8 (0.6)  2.6 (0.6) | 21.1 (3.9)  19.9 (3.5) | 71.8 (20.7)  66.3 (28.4) | 79.1 (22.3)  72.8 (17.6) | 55.6 (44.7)  56.4 (43.0) | 75.0 (28.6)  71.5 (27.7) | 1.7 (0.3)  1.7 (0.3) | 85.2 (11.8)  85.5 (9.2) |
| **Treatment**  By themselves (ScIg)  By others (IVIg) | 36  11 | 3.6 (0.7)  3.7 (0.4) | 2.6 (0.6)  2.8 (0.7) | 20.1 (3.4)  18.6 (3.6) | 70.5 (25.9)  57.6 (27.1) | 73.7 (18.0)  67.3 (17.9) | 60.7 (42.6)  36.4 (40.9) | 77.4 (23.9)  57.5^*^ (28.4) | 1.6 (0.3)  1.7 (0.3) | 84.5 (10.5)  84.0 (7.2) |
| **Cohabitation**  Living alone  Living with someone | 11  43 | 3.3 (0.7)  3.8^*^(0.6) | 2.9 (0.5)  2.52^*^ (0.6) | 19.6 (4.0)  20.1 (3.5) | 75.2 (31.6)  63.4 (26.9) | 70.5 (19.5)  74.4 (17.9) | 59.1 (43.7)  57.1 (42.5) | 73.8 (36.5)  70.6 (27.4) | 1.6 (0.3)  1.7 (0.3) | 79.3 (14.7)  86.2 (7.9) |
| **Employment**  Full time/Part time/Work at home  Unemployed | 37  17 | 3.7 (0.7)  3.6 (0.6) | 2.6 (0.6)  2.7 (0.7) | 20.8 (3.3)  18.1^**^(3.4) | 72.8 (25.1)  51.1^**^(28.0) | 76.9 (17.3)  64.5^*^ (19.0) | 69.1 (40.0)  26.6^**^(33.5) | 78.7 (25.0)  53.7^*^(29.9) | 1.6 (0.3)  1.8^**^(0.3) | 84.4 (9.9)  85.9 (10.1) |
| **Stressful events the last 2-3 months**  0-2  > 2 | 36  8 | 3.7 (0.7)  3.6 (0.6) | 2.6 (0.6)  2.9 (0.8) | 20.7 (3.3)  16.7^**^(3.7) | 68.8 (28.5)  59.5 (25.7) | 74.3 (18.8)  56.5^*^ (16.5) | 57.1 (41.8)  43.8 (50.0) | 74.6 (29.2)  50.0^*^(26.7) | 1.6 (0.3)  1.8 (0.4) | 85.2 (9.1)  85.9 (10.5) |

Levels of significance: *p=. 05, **p=. 01, ***p=. 001 (t-tests, two-tailed)

Abbreviations:

RPP Scale - Resources and pressures in the past. 1-5 scales. The higher score the higher level of pressures/strains or a better availability of resources.

QLI, Quality of Life Index: 0-30 scales. The higher score the better quality of life;

SF-36, Short Form-36 (Health-related quality of life): 0-100 scales. The higher score the higher functioning.

JCS, Jalowiec Coping Scale: 0-3 scales. The higher total score the more different coping strategies alternated.

NHS, Nowotny Hope Scale: 1-4 scales. The total score range as 29-116, with a high score indicating high hope.
